# Sun-Protective Behaviors of Student Spectators at Inter-school Swimming Carnivals in a Tropical Region Experiencing High Ambient Solar Ultraviolet Radiation

**DOI:** 10.3389/fpubh.2016.00168

**Published:** 2016-08-16

**Authors:** Denise Turner, Simone Lee Harrison, Nicole Bates

**Affiliations:** ^1^Skin Cancer Research Unit, Division of Tropical Health and Medicine, College of Public Health, Medical and Veterinary Sciences, James Cook University, Townsville, QLD, Australia; ^2^Anton Breinl Research Centre for Health Systems Strengthening, Australian Institute of Tropical Health and Medicine (AITHM), James Cook University, Townsville, QLD, Australia; ^3^Discipline of Pharmacy, Division of Tropical Health and Medicine, College of Medicine and Dentistry, James Cook University, Townsville, QLD, Australia

**Keywords:** skin cancer, swimming, child, sun-safety, ultraviolet radiation, ultraviolet protection factor, clothing, sun-protection

## Abstract

Skin cancer is the most common cancer in humans and Australia (particularly in Queensland) has the highest incidence globally. Sunlight is a known skin carcinogen and reflects off water, exacerbating the risk of sunburn. In 1988, the “SunSmart Program” was developed to promote sun-protection to Australian children. Within a decade, it evolved to include a voluntary national accreditation program for schools, known as the SunSmart Schools (SSS) Program. Additionally, in 2008, it became compulsory for primary schoolchildren attending Queensland government-funded schools to wear a shirt during all water-based activities, except when competing. We observed the proportion of student spectators from 41 Townsville (latitude 19.3°S) primary schools (65.9% SSS) wearing hats at inter-school swimming carnivals in 2009–2011 and 2015 and the proportion wearing a shirt. Overall, a median of 30.7% student spectators from each school wore a hat [max 46.2% (2009); min 18% (2015)] and 77.3% wore a shirt [max 95.8% (2009); min 74.5% (2015)], suggesting that hats are under-utilized. Students from non-government (private) schools were twice as likely as students from government schools to wear a hat (41 vs. 18.2% *p* = 0.003). Neither the hat nor the shirt-wearing behaviors of student spectators were significantly influenced by their school’s size (number of students), educational advantage, sun-protection policy score, or SunSmart status, indicating that other socioeconomic factors, not assessed here, may have influenced the results. Our findings suggest that the mandatory swim-shirt policy introduced in 2008 was very effective, especially initially. However, monitoring and feedback of results to schools may be needed to maintain high levels of compliance in the longer-term. Schoolchildren attending swimming carnivals should not rely on sunscreen or shade alone to protect against direct and reflected-sunlight, and need prompting to put a hat and shirt back on immediately after a race. This responsibility could be delegated to either a parent or a student prefect, if teachers are too busy to encourage and monitor sun-safety compliance among the students in their care.

## Introduction

Australia is an island nation; ~86% of Australians live within 50 km of the coast (1). Swimming is a national past-time and most children learn to swim as babies. Australians love the beach, fishing, swimming, surfing, playing sport, and being in the “great outdoors.” Generations of Australians were brought up playing outside; wore little sun-protection; and believed a tan signified good health (2, 3).

The Australian sun-loving culture paired with genetically susceptible Caucasian ancestry has resulted in Australia having among the highest rates of cutaneous melanoma (CM) and epithelial skin cancer in the world (4, 5). Solar ultraviolet radiation (UVR) is a known skin carcinogen and over-exposure, particularly during the childhood years, leads to the proliferation of melanocytic nevi (MN) (6–10), which are a risk factor for CM development (9, 11–13). Actinic keratoses, keratinocyte carcinomas, and CM are extremely common in the Queensland population and often develop on chronically sun-exposed areas of the face, ears, neck, and scalp (14–17).

Sun-protective clothing, especially garments manufactured according to the Australian and New Zealand clothing standard (AS/NZ 4399:1996) with tightly woven fabrics and high ultraviolet protection factor (UPF) ratings (18) provide a physical barrier between the skin and UVR and have been shown to slow the rate of development of MN (19, 20). Legionnaire, broad-brim and bucket hats protect the face, neck, and eyes better than caps and visors (21–24), with bucket hats being the most commonly worn style in north Queensland schools, followed by wide-brim hats (Turner and Harrison, unpublished data). Swimming garments that incorporate longer sleeves and pants present a practical form of sun-protection since, unlike sunscreen, they do not require reapplication (25). Pre-adolescent primary school-aged children have indicated that they find wet-suit type swimming clothes (sun-suits) which cover more of the skin than traditional swimwear visually appealing and would wear them if given the option (26). In spite of this, some schools in the high-risk UVR environment of north Queensland still make boys wear swimming briefs emblazoned with the school emblem, and girls wear a full-piece swimsuit in school colors with the same insignia when participating in swimming lessons and carnivals.

Most primary schoolchildren in Queensland get to participate in some water-based activities each year, such as swimming lessons and carnivals. These generally take place outdoors during the school day (typically between 8:30 a.m. and 3:00 p.m.) during or close to peak daily UVR times (27). At the beginning of the 2008 school year (primary school attendance follows a calendar year pattern in Australia from approximately late January to mid-December), the Queensland Government Department of Education and Training made it compulsory for students attending state-government-funded primary schools to wear either a swim-shirt or a T-shirt when participating in water-based activities and suggested that spectators adopt a range of sun-protection measures too (28). Students are only exempt from wearing a shirt while competing (29, 30). As UVR can both penetrate and reflect off water surfaces (31, 32), the unprotected skin of student competitors and spectators alike is exposed to overhead and reflected UVR that could be intercepted by clothing. Reflected UVR adds to the UVR dose received by spectators at swimming carnivals, making it unwise to rely on shade alone for protection; optimal sun-protection is achieved using several protective measures simultaneously, as exemplified by the Australian Cancer Council’s Slip (on a shirt), Slop (on some sunscreen), Slap (on a hat), Seek (shade), and Slide (on some sunglasses) campaign (33).

In 1988, after the internationally recognized “Slip! Slop! Slap!” campaign had been running in Australia for 8 years, the Cancer Council (formerly known as the Anti-Cancer Council of Victoria) developed the SunSmart program to improve the sun-protective behaviors of Australian children (34–37). The SunSmart Program evolved to include a national voluntary accreditation program known as the SunSmart Schools (SSS) Program that has been operating in Victorian primary schools since 1994, Queensland primary schools since 1999 and primary schools in the other Australian states and territories for over a decade (34). The SSS Program now also operates in Australian secondary schools, and has been adopted abroad by a number of other countries, including New Zealand, the United Kingdom, and South Africa (38–42). All Australian schools, regardless of school ownership [government (state-funded schools) or non-government (privately funded schools)] can apply to be SSS. Australian SSS are expected to comply with 12 sun-protection criteria concerning their sun-protection policy (43, 44). SSS are expected to encourage students to wear a T-shirt, sun-suit, or swim-shirt (also known as a rash-vest or “rashie”) when involved in swimming activities to give them extra protection in the water (43). Compliance with the behavioral expectations of the SSS program, such as hat and swim-shirt use, are not externally monitored at swimming carnivals or during any other curriculum-based outdoor activities; therefore, we present a unique look at how well schools are following through with their sun-protection policies. Our team has evaluated the sun-protection policies of Queensland primary schools (44–46) and identified the need for a school sun-protection intervention aimed at improving sun-protection policies and practices in Queensland primary schools. Data presented in this paper will be used as a baseline to evaluate changes in policies and behavior over time resulting from the intervention.

This observational study aimed to determine the proportion of primary school students (aged 5–12 years) wearing hats and shirts at inter-school swimming carnivals in the skin cancer prone population of Townsville, north Queensland, Australia (47, 48). Additionally, we suggest practical solutions to improve sun-protection among schoolchildren.

## Materials and Methods

### Participants

The sun-protective clothing-related behaviors exhibited by student spectators from 41 primary schools were observed at inter-school swimming carnivals held in Townsville each March (Early Autumn in the southern Hemisphere). Data were collected for 4 years between 2009 and 2015 (*n* = 10 carnivals; 2,932 students).

Townsville (latitude 19.3°S, longitude 146.8°E) is a coastal city in tropical north Queensland, Australia with a population of ~200,000 inhabitants who are primarily of European descent. This major regional center has a tropical climate with hot, humid summers, dry winters, and a high to extreme Maximum Daily UV-index (UVI), year round (49, 50). The mean UVI recorded on observation days was 10.1 ± 1.6 (51) with mean minimum and maximum temperatures of 22.7 ± 2.7°C and 31.7 ± 1.3°C, respectively (49, 50).

### Procedure

Schools present at any of the inter-primary school swimming carnivals held in Townsville in 2009, 2010, 2011, and 2015 were included in the study. At each of the 10 carnivals, an experienced observer (drawn from a pool of 3 observers; i.e., the authors) counted the proportion of students in a school’s designated spectator area who were wearing a hat. The process was then repeated to determine the proportion of primary school students from the same school who were wearing a shirt of any description (swim-shirt; T-shirt; school shirt; sun-suit, etc.). The entire process was repeated for each school in attendance. Observations were made discretely and the purpose of the study was not discussed with individuals to avoid influencing their behavior. Observations were conducted from inside the pool complex during the first hour of the carnival, well after all students had time to settle into their school’s designated viewing area. Students were assigned to the hat “present” or “absent” group separately to being assigned to the shirt “present” or “absent” group since it was too slow and difficult for a single observer to accurately record hat and shirt usage simultaneously for each student spectator. Students were not always seated in their designated school area; therefore, students were only included in these observations if the school they attended was identifiable by location or uniform. For example, a student attending “School A” may have been observed while in “School B’s” seating area but was identifiable as a “School A” student because they were wearing the uniform or hat of that school. Conversely, a student may have been excluded from observation if seated on the boundary of two school areas such that the school they attended was not identifiable from their clothing (e.g., not wearing a school hat or shirt).

The SunSmart status of each school was confirmed by Cancer Council Queensland, while demographic information [e.g., school ownership; location; student enrollments; “Index of community socio-educational advantage” (ICSEA)] was obtained from links provided on the Queensland Government website (52) and the Australian “My School” website (53). Each school’s sun-protection policy was independently evaluated against 12 pre-determined criteria and a total score was assigned as described previously (44). The distribution of demographic characteristics is shown in Table 1.

**Table 1:** **Demographic characteristics of the 41 schools who attended at least one of the inter-primary-school swimming carnivals held in Townsville, Queensland in 2009, 2010, 2011, and 2015, stratified by SunSmart status)**.

School characteristic		All schools (*N* = 41) *N* (%)	SunSmart schools (SSS)^a^ (*N* = 27) *N* (%)	Non-SSS (*N* = 14) *N* (%)	*p*-Value^f^
SunSmart School^a^	Yes	27 (65.9)	–	–	–
	No	14 (34.1)	–	–	
School type	Primary^b^	35 (85.4)	23 (85.2)	12 (85.7)	1.000 (Exact)
	Combined^c^	6 (14.6)	4 (14.8)	2 (14.3)	
School ownership	Government	26 (63.4)	16 (59.3)	10 (71.4)	0.443
	Non-government	15 (36.6)	11 (40.7)	4 (28.6)	
Sun-protection policy score^d^	≤ Median score (0–2)	21 (51.2)	12 (44.4)	9 (64.3)	0.228
	> Median score (3+)	20 (48.8)	15 (54.6)	5 (35.7)	
School size	Small (≤399 students)	17 (41.5)	11 (40.7)	6 (42.9)	0.668
	Medium (400–799 students)	15 (36.6)	9 (33.3)	6 (42.9)	
	Large (≥800 students)	9 (21.9)	7 (25.9)	2 (14.3)	
ICSEA group^e^	ICSEA ≤1000	35 (85.4)	21 (77.8)	14 (100.0)	0.079 (Exact)
	ICSEA >1000	6 (14.6)	6 (22.2)	0 (0.0)	

*^a^SunSmart status was verified by contact with the Cancer Council Queensland, as at December 2012*.

*^b^Primary school starts at age 5 (prep year) and continued until Grade 7 (age 12 years) in Queensland up until 2015 when grade 7 became the first year of secondary schooling; first 8 years of formal education*.

*^c^Combined schools enroll students for their entire formal education (Prep – Grade 12)*.

*^d^Total score attained by these 41 schools when their sun-protection policies were independently evaluated against pre-determined criteria [maximum possible score was 12 (44)]*.

*^e^The index of community socio-educational advantage (ICSEA) is calculated using student family background data to determine the level of educational advantage students bring to their studies. The average ICSEA value is set at 1000 with values ranging from 500 (extremely educationally disadvantaged backgrounds) to 1300 (students from highly educated families)*.

*^f^*P*-value based on Chi-squared test (or two-sided Fisher’s Exact test if ≥25% of cells have an expected frequency of ≤5); *p* < 0.05 statistically significant*.

### Data Analysis

Hat and shirt-wearing rates were calculated for each school by combining observations across 4 years of data. Hat and shirt-wearing proportions were summed across years, and described using median values together with inter-quartile range (IQR) and range (minimum and maximum values) as the data were skewed. Non-parametric Mann–Whitney and Kruskal–Wallis tests were used to assess differences in the median proportion of students wearing a hat and the median proportion of students wearing a shirt according to SunSmart status and the other demographic characteristics described in Table 1. Differences in student denominators for hat- and shirt-wearing proportions are attributable to students moving around the venue during the carnivals (e.g., students might have been in the pool, bathrooms, away from their designated school areas, etc.) since hat-wearing observations preceded shirt-wearing observations.

## Results

The proportion of student spectators from each school observed wearing hats ranged from 0 to 83.3% with a median value of 30.7% (Table 2). Students from non-government schools were twice as likely to be seen wearing a hat as government primary school students (41.0 vs. 18.2%; *p* = 0.003). Average ICSEA scores (continuous variable) were higher for non-government schools compared with government schools (977.9 vs. 918.2; *p* = 0.009), suggesting that students from non-government schools may have a socio-education advantage. Student hat-wearing rates did not differ significantly according to any of the other demographic characteristics considered (SunSmart status, sun-protection policy score, and school size), except for school type, where the difference in hat-wearing rates between combined primary-secondary schools (43.9%) and dedicated primary schools (23.1%) was borderline significant (*p* = 0.051; Table 2).

**Table 2:** **The median (IQR); range (*n*) proportion of student spectators per school observed wearing hats and shirts while attending inter-primary-school swimming carnivals in Townsville, Australia over 4 years of observations (2009–2011 and 2015)**.

		Proportion (%) of students at each school wearing a HAT based on *n* = 2,916 observations conducted for a sample of *N* = 41 schools		Proportion (%) of students at each school wearing a SHIRT based on *n* = 2,932 observations conducted for a sample of *N* = 41 schools	
			
		Median% (IQR); range% (*n*)	*P*-value	Median% (IQR); range% (*n*)	*P*-value
All schools (*N* = 41)		30.7 (13.2, 46.7); 0.0–83.3		77.3 (70.0, 85.9); 41.7–100.0	
School characteristic SunSmart school^a^	Yes (*N* = 27) No (*N* = 14)	36.3 (13.0, 48.8); 5.0–83.3 (2,206) 23.6 (12.3, 37.1); 0–81.0 (710)	0.422	77.3 (71.0, 85.0); 54.3–100.0 (2,236) 76.2 (57.8, 91.8); 41.7–100.0 (696)	0.559
School ownership	Government (*N* = 26) Non-Government (*N* = 15)	18.2 (9.8, 37.9); 0.0–72.2 (1,592) 41.0 (30.3, 57.9); 13.3–83.3 (1,324)	0.003	77.5 (69.8, 85.9); 41.7–100.0 (1,577) 76.8 (70.9, 86.8); 54.3–100.0 (1,355)	0.989
Sun-protection policy score^b^	≤ Median score (0–2) (*N* = 21) > Median score (3+) (*N* = 20)	23.1 (12.5, 43.1); 0.0–83.3 (1,223) 36.2 (15.7, 47.9); 5.0–81.0 (1,693)	0.348	77.6 (66.0, 90.7); 41.7–100.0 (1,247) 76.4 (70.0, 83.2); 43.8–100.0 (1,685)	0.361
School size	Small (≤399 students) (*N* = 17) Medium (400–799 students) (*N* = 15) Large (≥800 students) (*N* = 9)	14.3 (9.7, 47.4); 0.0–83.3 (718) 36.3 (20.7, 41.0); 9.1–54.2 (1,242) 36.9 (22.7, 49.8); 9.1–57.9 (956)	0.228	85.0 (69.4, 100.0); 41.7–100.0 (725) 75.9 (58.6, 83.0); 43.8–92.3 (1,234) 74.7 (71.1, 77.5); 70.0–86.8 (973)	0.142
ICSEA group^c^	ICSEA ≤1000 (*N* = 35) ICSEA >1000 (*N* = 6)	30.3 (13.0, 38.1); 0.0–83.3 (2,345) 49.4 (12.2, 53.1); 6.0–57.9 (571)	0.319	75.9 (69.3, 85.0); 41.7–100.0 (2,351) 82.2 (75.4, 91.4); 71.1–100.0 (581)	0.209
School type	Primary^d^ (*N* = 35) Combined^e^ (*N* = 6)	23.1 (11.9, 41.0); 0.0–83.3 (2,397) 43.9 (34.2, 63.7); 30.3–81.0 (519)	0.051	77.5 (70.0, 85.0); 41.7–100.0 (2,391) 74.0 (57.5, 90.1); 54.3–100.0 (541)	0.679

*^a^SunSmart status was verified by contact with the Cancer Council Queensland, as at December 2012*.

*^b^Total score attained by these 41 schools when their sun-protection policies were independently evaluated against pre-determined criteria [maximum possible score was 12 (44)]*.

*^c^The index of community socio-educational advantage (ICSEA) is calculated using student family background data to determine the level of educational advantage students bring to their studies. The average ICSEA value is set at 1000 with values ranging from 500 (extremely educationally disadvantaged backgrounds) to 1300 (students from highly educated families)*.

*^d^Primary school starts at age 5 (prep year) and continued until Grade 7 (age 12 years) in Queensland up until 2015 when grade 7 became the first year of secondary schooling; first 8 years of formal education*.

*^e^Combined schools enroll students for their entire formal education (Prep – Grade 12)*.

The proportion of student spectators observed wearing a shirt ranged from 41.7% for some schools to 100% in others, with a median of 77.3%. Shirt-wearing rates did not differ significantly according to SunSmart status or any of the other demographic characteristics examined (Table 2).

Hat-wearing rates were higher among non-government (privately funded) SSS than government-run SSS (48.8 vs. 17.5%; *p* = 0.005) and large and medium SSS (45.2 vs. 38%) compared with small SSS (13%; *p* = 0.048). No other statistically significant differences in hat-wearing or shirt-wearing proportions were found when the other remaining school characteristics were explored within SunSmart status or vice versa (Table 3).

**Table 3:** **Median (IQR); range (*n*) of the proportion of student spectators observed wearing hats and shirts while attending at least one inter-primary-school swimming carnival during the 4 years of observations carried out 2009–2011 and 2015 are shown stratified by SunSmart status within each of the school characteristics considered**.

		Proportion (%) of students at each school wearing a HAT based on *n* = 2,916 observations conducted for a sample of *N* = 41 schools				Proportion (%) of students at each school wearing a SHIRT based on 2,932 observations conducted for a sample of *N* = 41 schools			
School characteristic		SunSmart^a^ school (SSS) (*N* = 27)		Non-SSS (*N* = 14)		SSS^a^ (*N* = 27)		Non-SSS (*N* = 14)	
			
		Median% (IQR); range (*n*) in (*N* schools)	*p*-Value within SSS ↓	Median% (IQR); range (*n*) in (*N* schools)	*p*-Value within Non-SSS↓	Median% (IQR); range (*n*) in (*N* schools)	*p*-Value within SSS↓	Median% (IQR); range (*n*) in (*N* schools)	*p*-Value within Non-SSS↓
School ownership	Government, *p*-value^¥^ →	17.5 (10.3, 38.0); 5.0–54.2 (975) (*N* = 16)	0.005 ↓ 0.979→	19.9 (9.1, 37.1); 0.0–72.2 (617) (*N* = 10)	0.539↓	80.1 (70.3, 87.6); 60.9–100.0 (970) (*N* = 16)	0.645↓ 0.363→	76.2 (53.0, 85.4); 41.7–100.0 (607) (*N* = 10)	0.539↓
	Non-government, *p*-value^¥^ →	48.8 (33.9, 57.9); 16.1–83.3 (1,231) (*N* = 11)	0.343→	29.3 (15.8, 69.6); 13.3–81.0 (93) (*N* = 4)		76.8 (71.1, 83.2); 54.3–100.0 (1,266) (*N* = 11)	0.949→	79.3 (58.4, 100.0); 58.3–100.0 (89) (*N* = 4)
Sun-protection policy score^b^	≤ Median score (0–2), *p*-value^¥^ →	35.1 (13.3, 49.5); 6.0–83.3 (837) (*N* = 12)	0.905↓ 0.247→	15.0 (9.1, 30.5); 0.0–72.2 (386) (*N* = 9)	0.083↓	80.7 (71.7, 91.3); 54.3–100.0 (857) (*N* = 12)	0.373↓ 0.862→	77.6 (57.2, 94.5); 41.7–100.0 (390) (*N* = 9)	0.797↓
	> Median score (3 +), *p*-value^¥^ →	36.9 (11.1, 48.8); 5.0–58.0 (1,369) (*N* = 15)	0.745→	35.5 (23.2, 59.6); 15.6–81.0 (324) (*N* = 5)		76.8 (70.9, 83.2); 67.9–94.4 (1,379) (*N* = 15)	0.306→	69.3 (51.2, 89.0); 43.8–100.0 (306) (*N* = 5)
School size	Small (≤ 399 students), *p*-value^¥^ →	13.0 (8.3, 22.7); 5.0–83.3 (607) (*N* = 11)	0.048↓ 0.301→	30.0 (10.0, 74.4); 0.0–81.0 (111) (*N* = 6)	0.385↓	85.0 (70.9, 94.4); 60.9–100.0 (616) (*N* = 11)	0.351↓ 0.884→	92.1 (54.2, 100.0); 41.7–100.0 (109) (*N* = 6)	0.459↓
	Medium (400–799 students), *p*-value^¥^ →	38.0 (28.5, 49.4); 10.0–54.2 (811) (*N* = 9)	0.088→	27.5 (14.0, 36.2); 9.1–38.1 (431) (*N* = 6)		77.5 (70.6, 83.5); 54.3–92.3 (810) (*N* = 9)	0.224→	64.0 (53.0, 80.8); 43.8–89.0 (424) (*N* = 6)
	Large (≥800 students), *p*-value^¥^ →	45.2 (33.9, 51.5); 30.3–57.9 (788) (*N* = 7)	0.056→	12.1 (9.1, –); 9.1–15.0 (168) (*N* = 2)		73.9 (71.0, 77.3); 70.0–86.8 (810) (*N* = 7)	0.5→	76.2 (74.7, –); 74.7–77.6 (163) (*N* = 2)
ICSEA group^c^	ICSEA ≤ 1000, *p*-value^¥^ →	33.9 (12.5, 43.1); 5.0–83.3 (1,635) (*N* = 21)	0.345↓ 0.538→	23.7 (12.3, 37.1); 0.0–81.0 (710) (*N* = 14)	–	75.9 (70.5, 84.5); 54.3–100.0 (1,655) (*N* = 21)	0.175↓ 0.702→	76.2 (57.8, 91.8); 41.7–100.0 (696) (*N* = 14)	–
	ICSEA > 1000, *p*-value^¥^ →	49.4 (12.2, 53.1); 6.0–57.9 (571) (*N* = 6)		–		82.2 (75.4, 91.4); 71.1–100.0 (581) (*N* = 6)		–
School type	Primary^d^, *p*-value^¥^→	33.9 (11.9, 48.4); 5.0–83.3 (1,739) (*N* = 23)	0.243↓ 0.362→	19.4 (10.2, 35.3); 0.0–72.2 (658) (*N* = 12)	0.132↓	77.5 (71.0, 85.0); 60.9–100.0 (1,745) (*N* = 23)	0.448↓ 0.420→	76.2 (56.7, 87.8); 41.7–100.0 (646) (*N* = 12)	0.659↓
	Combined^e^ *p*-value^¥^→	43.9 (31.8, 56.3); 30.3–57.9 (467) (*N* = 4)	0.8→	58.2 (35.5, –); 35.5–81.0 (52) (*N* = 2)		74.0 (58.5, 84.3); 54.3–86.8 (491) (*N* = 4)	0.8→	79.3 (58.6, –); 58.6–100.0 (50) (*N* = 2)

*First *p*-value compares hat-wearing proportions across categories of a demographic characteristic within a single SunSmart status group (↓direction of comparison is downwards, i.e., within SunSmart status)*.

*^¥^Second *p*-value compares hat-wearing proportions across SunSmart status groups within a single strata of a demographic characteristic (→direction of comparison is across, i.e., within a single category of demographic characteristic). All *p*-values comparing hat-wearing proportion at SSS compared to Non-SSS; and shirt-wearing proportion at SSS compared to Non-SSS produced non-significant results (*p* > 0.05)*.

*^a^SunSmart status was verified by contact with the Cancer Council Queensland, as at December 2012*.

*^b^Total score attained by these 41 schools when their sun-protection policies were independently evaluated against pre-determined criteria [(maximum possible score was 12 (44)]*.

*^c^The index of community socio-educational advantage (ICSEA) is calculated using student family background data to determine the level of educational advantage students bring to their studies. The average ICSEA value is set at 1000 with values ranging from 500 (extremely educationally disadvantaged backgrounds) to 1300 (students from highly educated families)*.

*^d^Primary school starts at age 5 (prep year) and continued until Grade 7 (age 12 years) in Queensland up until 2015 when grade 7 became the first year of secondary schooling; first 8 years of formal education*.

*^e^Combined schools enroll students for their entire formal education (Prep – Grade 12)*.

Furthermore, the proportion of student spectators observed wearing a hat appeared to decline over the study, from a median of 46.2% in 2009 to 18.0% in 2015. A similar temporal trend was also evident for the proportion of student spectators observed wearing a shirt which declined from a median of 95.8% in 2009 to 74.5% by 2015 (Table 4).

**Table 4:** **Median (IQR); range of student spectator hat-wearing and shirt-wearing proportion at Townsville inter-primary-school swimming carnivals during the 4 years of observations carried out 2009–2011 and 2015 are shown stratified by year**.

Year	Median% of students wearing a hat	Median% of students wearing a shirt
2009	46.2 (39.2,56.0); 36.4–66.7	95.8 (80.6, 96.8); 77.1–97.4
2010	36.7 (16.2, 51.3); 6.9–80.0	80.6 (67.3, 90.4); 35.4–97.2
2011	27.4 (12.7, 39.3); 0.0–100.00	78.0 (66.1, 88.5); 40.0–100.0
2015	18.0 (7.7, 42.5); 0.0–76.9	74.5 (55.9, 90.0); 0.0–100.0

## Discussion

To our knowledge, this is the first report to comment on the sun-protective behaviors of student spectators at school swimming carnivals in Australia. We found student hat-wearing rates at Townsville inter-primary-schools swimming carnivals to be poor; a concern in this skin cancer prone population (47, 48). More student spectators were seen wearing a shirt (median 77.3%) than a hat (median 30.7%), confirming our earlier assertions (54, 55) and the anecdotal reports of others (56) that hats are vastly under-utilized by schoolchildren in Queensland. The proportion of student spectators who were observed wearing a shirt was not associated with any of the socio-demographic characteristics we considered, whereas hat-wearing rates differed significantly between government and non-government schools, and to a lesser extent, by school type (primary vs. combined primary–secondary schools). One possible explanation is that of positive role-modeling, where younger students mimic good sun-protective behaviors that are modeled for them by older students during their schooling. Assuming that this is true and that these behaviors become habitual, this phenomenon could result in primary schoolchildren from combined primary–secondary schools exhibiting better hat-wearing compliance at inter-school swimming carnivals than schoolchildren from traditional primary schools. However, we did not collect data describing hat-use among north Queensland secondary school students to support this hypothesis, and others consistently report poor hat-use among secondary students, both within Australia and abroad (2, 56, 57) making this explanation less plausible. It is worth noting that only six of the 41 schools in our study population were “combined” schools, and that all of them were non-government schools. Thus, it is difficult to separate out the influence of school type (i.e., primary vs. combined schools) and school ownership (i.e., government-funded vs. non-government schools) in the present study.

The ICSEA scores of non-government (privately funded) schools were higher than those of government schools in the present study, suggesting that non-government schoolchildren in Townsville have a socioeconomic advantage over children attending government-funded schools in the same district. This may include better access to financial resources (e.g., sufficient discretionary household income to replace a lost school hat) or having more highly educated parents. The latter could potentially result in non-government schoolchildren receiving better education about sun-protection at home than their government-school counterparts. While socioeconomic advantage may be a plausible explanation for hat-wearing being more prevalent among non-government schoolchildren, it fails to explain why the same was not true for wearing a shirt. In fact, we found that swim-shirt rates were almost identical for the non-government (76.8%) and government schools (77.5%) observed in the present study.

When examining temporal trends in shirt-usage among student spectators, we found that shirt-wearing compliance was highest at the beginning of the study in 2009. The “almost perfect” result of 95.8% was achieved soon after the “no shirt, no swim” rule (28), was introduced in Queensland, making it compulsory for primary schoolchildren attending state-government-funded schools to wear a shirt during school water-based activities (except when competing). This result demonstrates just how effective the mandatory swim-shirt policy was at the time of its implementation (29, 30). Anecdotal evidence from the newsletters of non-government schools in the study area suggests that implementation of the swim-shirt policy was not confined to government schools, with a number of non-government schools in Townsville also stating their intention to adopt the “no shirt, no swim” rule (Harrison, unpublished data). This seems to be a plausible explanation for the similarly high shirt-wearing rates that were observed for most schools, irrespective of whether they were government or non-government-run facilities.

Consistent with the mandatory swim-shirt policy hypothesis, we also documented a substantial decrease of 15.2% in shirt-wearing rates between carnivals held in March 2009 (~13-months after introduction of the swim-shirt policy) and those held in March 2010 (25-months post-introduction). Shirt-usage rates continued to decline in the years following 2010, albeit at a slower pace, reaching a minimum of 74.5% by 2015; the final year of the study. This phenomenon is most likely due to a decline in media interest, and possibly also diminished departmental communication with schools about the mandatory swim-shirt policy in the years following its introduction.

SunSmart guidelines also recommend that students wear sun-protective clothing, such as T-shirts or rash-vests when involved in swimming activities and that wet shirts be replaced with dry ones when exiting the pool (43). However, similar proportions of children from SSS and Non-SSS were observed wearing a shirt (77.3 and 76.2%, respectively) in this study, suggesting that the SSS program had little, if any, additional impact on swim-shirt compliance in tropical north Queensland schools.

Student spectators and competitors alike should wear shirts to protect their torso from unnecessary UVR since it is reflected from pool water surfaces and ~60% can penetrate into pool water (31, 32). Drag from shirts can be reduced substantially for competitors if properly fitting rash-vests are worn, and competitive swimmers have actually benefited from reduced drag by wearing all-in-one elastane suits (58). Given that swim-shirt use is optional for competing students, in this climate at this time of year [average recorded UV index for March 2009, 2010, 2011, and 2015 was 9.7 (51)], students can easily exceed the daily UVR exposure limit while lining up several races ahead of their own (often for more than 6 min) event with much/all of their torso exposed. If a shirt is not worn during an event, at the very least, it should be worn up to the time of the event and put back on immediately after exiting the pool.

In 1996, Australia pioneered the relative ranking (UPF) of the sun-protective capabilities of clothing based on the transmission of UVR though fabric. The UPF rating is printed on the swing tags of sun-protective clothing sold in Australia to guide consumers in purchasing sun-protective garments, such as swim-shirts for themselves and their children. However, as minimum body surface coverage is not specified in the current standard (AS/NZS 4399:1996) (59, 60), some swimwear manufacturers have taken advantage of this loop-hole to market elastane (Lycra^®^) bikinis with UPF 50+ swing tags attached (59). Our randomized controlled trial (RCT) demonstrated that sun-protective clothing that covers more body surface area (BSA) can reduce the development of MN in young children and subsequent melanoma risk (19, 20). Consequently, considerable effort has been invested recently to revise the Australian and New Zealand Standard for sun-protective clothing to address this issue (59, 61). Sun-protective clothing made of high UPF fabric with elbow-length sleeves was well tolerated by children in our previous RCT and prevented a significant proportion of new MN developing on the upper arms (19, 20). Furthermore, co-author (Simone Lee Harrison) has successfully trialed a swim-shirt loan scheme in several north Queensland primary schools in recent years. Preliminary results of this translational research project suggest that it may offer a novel and cost-effective solution to providing schoolchildren with equal access to good quality, long-sleeve sun-protective shirts for use during curriculum-based water activities (Harrison, unpublished data).

UVR is a skin carcinogen and contributes to eye and surrounding tissue damage, age-related cataracts, corneal degenerative changes, and possibly age-related macular degeneration (62, 63). The risk of over-exposure is further exacerbated at outdoor aquatic events as UVR reflects off water, further increasing an individual’s exposure; making it especially important that children use multiple methods of sun-protection, including hats, shade, sunscreen, and sunglasses to protect skin on the face and neck in aquatic environments (32). In response to the dangers associated with over-exposure, the International Radiation Protection Association recommends that an individual’s daily UVR exposure does not exceed 30 J m^−2^ (64). However, recent research shows that during summer, Queensland teachers can exceed their weekly UVR dose in a single day between 8:30 a.m. and 3:15 p.m. (average daily exposure: 115 J m^−2^) since they are required to spend a considerable amount of time outdoors during peak UVR exposure times supervising students during lunch breaks, physical education classes, sporting events, etc. (65, 66). Additionally, Downs and Parisi (67) report considerable variability within student UVR dose during the school day at South East Queensland; the median student exposure during a typical school day was found to be 1.6 SED (standard erythema dose; 1 SED = 100 J/m^2^ of erythemally effective UV exposure) while students at school swimming carnivals were exposed to almost 50 SED. On a clear day, when the UVI is 12–14 (a typical Spring/Summer day for the study location), it takes only 6–7 min for a unprotected individual to receive their daily UVR limit (64). Individuals can easily determine the appropriate level of sun-protection required for their environmental conditions via the Australian Radiation Protection and Nuclear Safety Agency website (provides up-to-the-minute UVR reports for Australian capital cities) (51) or via the Australian Cancer Council’s mobile phone application (uses the Bureau of Meterology to report UVI) (68).

Hats shade the face and neck from excessive sun exposure (24). Queensland Government schools and SunSmart accredited schools have sun-safety guidelines that stipulate that students are expected to wear a hat when outdoors (34, 69). However, evidence from the present study (18% hat-wearing rates in 2015) and research conducted previously by our team (54, 55) suggests that hats are still under-utilized by primary schoolchildren living in north Queensland’s intense ambient UVR climate.

We expected a significantly higher proportion of students from SSS than non-SSS to be observed wearing a hat, since SunSmart guidelines specify that all primary schoolchildren should wear a broad-brimmed (≥7.5 cm brim), legionnaire or bucket hat (≥6 cm brim, deep crown) when outside (43). The difference in median hat-wearing proportions between SSS and non-SSS was 12.7%, but was not substantial enough to reach statistical significance in the present study of 41 schools (SSS 36.3 vs. 23.6%; *p* = 0.422). When the results were further stratified, some hat-wearing rates seemed higher for SSS than for non-SSS. For example, a higher proportion of students attending large SSS wore hats compared to their peers at large non-SSS (45.2 vs. 12.1%; *p* = 0.056). This result was only borderline significant, but had limited statistical power to detect a difference as it was based on just nine schools (only two of which were large non-SSS). Similarly, while the effect of SunSmart status on hat-wearing within government schools was negligible (2.4% difference; *p* = 0.979), the difference in hat-wearing proportions across categories of SunSmart status in non-government (privately funded) schools was almost 20% (non-government SSS 48.8% vs. non-government non-SSS 29.3%; 0.343). Again, this failed to reach statistical significance, most likely due to the small sample size (there were only four non-government non-SSS in the study region) and the lack of statistical power. Although SunSmart status may have some degree of influence over spectator hat-wearing compliance that was difficult to quantify in this relatively small study of 41 schools, it was apparent that school ownership (a likely indicator of socioeconomic status) exerted more influence over hat-wearing prevalence than SunSmart status, as suggested by the finding that significantly more students from non-government SSS than government SSS were observed wearing a hat (48.8 vs. 17.5%, respectively; *p* = 0.005).

Accordingly, we suggest that while SunSmart status may have some influence on hat-wearing compliance among primary student spectators compliance, the hat-wearing proportions observed for the 27 SSS in this study were far from remarkable at a median of 36.3%. This is a concern since these schools are provided with sun-safety resources; encouraged to develop a comprehensive school sun-protection policy; and make a written commitment to improve sun-safety in their school environment. Considered as a whole, these observations demonstrate that the expectations of the SSS Program are not being closely adhered to in this high-risk population, since most of the students we observed at SSS and non-SSS alike failed to wear their hat. Consistent with the suggestions of key stakeholders about increasing the external accountability of schools (70) our research group is trialing a school-based sun-protection program that monitors sun-protection compliance and feeds this information to individual primary schools.

Our results also suggest that the sun-protective behaviors of primary schoolchildren from this skin cancer prone region declined over the period of the study. Almost two decades have passed since the SSS Program was introduced in Queensland, and 8 years have passed since it became mandatory for primary schoolchildren from government schools in Queensland to wear a shirt during all water-based activities except swimming races. Consequently, all Queensland schools catering to primary students should be aware of the dangers associated with over-exposure to UVR, yet it seems that the sun-safety message is failing to reach a significant proportion of its target audience. It is not known whether this is because the message has little or no effect, or whether teachers and students from primary schools under-estimate the long-term effects of over-exposure to UVR. We could not measure sunscreen application cost-effectively as part of the baseline phase of this trial, so it is possible that some of the students who were observed were wearing sunscreen, however, it is not advisable to rely on sunscreen alone since it does not provide full protection; needs to be applied 20 min before going outdoors and reapplied every 2 h (more frequently when participating in water-based activities) (71). More prompting, education, guidance, and monitoring may be required to improve hat-wearing compliance at school sporting events since it is likely that sun-protective behaviors lapse as spectators settle into watching events or fail to retrieve their hat (and/or shirt if swimming) after competing in an event. Additionally, numerous schools and students are present at swimming events and school staff may be preoccupied with organizing events, recording results and preparing students for races, and forget to prompt the children they are supervising to put their hats and shirts back on. All of the schools that we observed had one or more parents present at the carnival. Therefore, this problem could be alleviated by having each school assign one such parent to champion sun-protection (or several parents could fill this role in succession) for the duration of the carnival. Alternatively, staff could charge a school prefect or sports captain with this responsibility. This would ensure that someone is focused on supervising the sun-safety practices of students, and prompting them to put their hats and shirts back on after an event, and to apply sunscreen and return to shaded areas where available. Senior primary schoolchildren could be encouraged to conduct their own observations of sun-protective behavior; use their mathematic skills to interpret the data; and encouraged to present their findings to their class and school staff and management using graphs and charts, etc. This would also benefit the students by demonstrate to them how skills learnt in the classroom can be applied to everyday life. Additionally, students could help institute change in sun-protective behaviors by taking periodic photos of their school spectator areas then retrospectively calculate hat- and shirt-wearing proportions and this information could be used by schools to commend/reward sports houses/teams who consistently demonstrate appropriate sun-protective behavior. Recent discussions with school principals involved in our ongoing interventional research have highlighted some of the innovative strategies that they have used to improve sun-protection compliance at outdoor sporting events. One non-government school (Annandale Christian College, Townsville, QLD, Australia) provides students with adhesive disposable wristbands which are “ticked” every time students re-apply sunscreen at the “sunscreen station” provided by the school. Students cannot participate in their nominated event unless their wristband indicates they have applied sunscreen hourly. This strategy could be adapted for use at swimming carnivals by using waterproof adhesive wristbands suitable for aquatic use and by using stickers instead of indelible pen markings each time sunscreen is applied. Two Townsville schools also rescheduled their swimming carnivals to the evening to avoid excessive sun exposure, however one school experienced poor student and parental attendance after doing so, and had to revert back to holding their swimming carnival during school hours. Rescheduling outdoor activities to avoid peak UVR periods is advantageous, but can be problematic in tropical locations where sun-protection is often required (i.e., the UVI is 3 or above) from 8:30 a.m. until 3.30 p.m. since this would mean school staff, students, and parents would be required to attend outside usual school hours.

The aforementioned approaches could be used by primary schools for other outdoor sports carnivals and even excursions and are synonymous with the views shared at a workshop attended by teachers, education policy makers, and other key stakeholders from Queensland during which, strong support was shown for monitoring sun-protection compliance, increasing external accountability, and working toward cultivating internal champions to assist with the implementation of sun-protection in Queensland schools(70). Since this report was published (70), the Queensland Government Department of Education and Training has introduced policies governing the attendance of Queensland government schools at swimming carnivals and other aquatic activities. Teachers arranging for students to attend these events are expected to conduct a Curriculum Activity Risk Assessment to manage all foreseeable risks (72) and follow these guidelines for swimming carnivals (73) These guidelines currently mention that the event must comply with the school’s sun-safety strategy in regard to competitors and spectators (69); state that adequate shade should be available; and specify that, “for events longer than 2 h, provide regular reminders to stay in the shade as much as possible, wear hats and sunglasses, re-apply sunscreen,….” These guidelines are quite explicit and suggest designating roles to adults, such as a first aid officer and lifeguard. These guidelines could be improved by suggesting that a designated sun-safety officer be assigned for swimming carnivals and mention that a parent or student prefect could fill this role. Policy guidelines, such as this, especially once refined, may provide a useful model worthy of adoption in school communities in high ambient UVR environments in the northern Hemisphere.

In-service education for school staff and education policy makers in high-risk regions might also be useful in making them aware of how quickly children can burn in regions with high levels of ambient UVR. They also need to be made aware that it is possible for students to sustain a sunburn even while in the shade in aquatic settings, if personal sun-protection is not used, as the reflectance of UVR off the surface of water can be substantial. It is especially important to remain vigilant about personal sun-safety since UVR is not visible to the naked eye; making it easy to dismiss. The causative link between UVR over-exposure and skin cancer development is well established, yet our schoolchildren seem to be at risk of over-exposure. To better understand why observed sun-protective behaviors were inadequate, it would be advantageous to meet with school staff, parents, and caregivers to discuss the value of multiple methods of personal sun-protection; and learn why observed behaviors were poor. Perhaps the sun-safety message is misunderstood; the dangers of over-exposure to UVR are under-estimated; sun-protective behaviors are perceived as inconvenient; or schoolchildren consider school hats and swim-shirts/t-shirts to be “uncool,” therefore, chose not to wear them.

This unique research presents data obtained from direct observations of shirt and hat-wearing behaviors at primary school swimming carnivals. Our research is limited by the small number of schools operating in the region (*n* = 41) and the associated lack of statistical power. As students could not be filmed or photographed due to ethical restrictions, it is possible that the total number of individuals and proportion wearing hats and shirts may have been slightly over or under-reported. However, such information bias would be similar for all schools and result in a bias toward the null in comparative analyses. Our research is strengthened by the use of observational data that were collected without informing study participants of the nature of the research. Collecting data this way provides a truer representation of student sun-protective behaviors that were not influenced by our presence. While we wanted to present data on the sun-protection practices of adult role-models as well, in practice, we found it difficult to accurately group the adults we observed (particularly parents and other spectators) with specific schools since adults did not always sit in designated school areas. Future reports may benefit from grouping all observed adults together rather than categorizing adults according to individual schools. Student sun-protective behaviors may be influenced by the same behaviors of all staff, parents, caregivers, and other adult spectators present at a swimming carnival (or other school sporting event) and not only by the adults associated with their particular school.

## Conclusion

Sun-protection during the childhood years is important for reducing the risk of developing skin cancer later in life. We found that primary school student hat-wearing rates at inter-school swimming carnivals in a region with intense ambient UVR and high skin cancer rates were poor and that shirt-wearing rates, while quite good, could still be improved. School demographics, including student enrollment numbers, sun-protection policy evaluation score, and SunSmart status were not found to remarkably impact sun-protective behaviors. The value of using multiple forms of sun-protection at school swimming carnivals needs to be emphasized, especially as spectators and competitors are exposed to both reflected and direct UVR. A single form of sun-protection rarely provides adequate protection against over-exposure to sunlight under these circumstances and one can receive their daily UV exposure limit in a matter of minutes when insufficiently protected, particularly in tropical and sub-tropical locations.

## Ethics Approval

The study was approved by James Cook University (approvals H3365; H5279; H6088), Education Queensland (ref 11/54273; 550/27/1112; 550/27/1497), and the Catholic Diocese of Townsville (2011-06; 2015-07).

## Author Contributions

DT – Data collection, data analysis, manuscript preparation, and submission. Dr. SH – Data collection and manuscript preparation. NB – Data collection and manuscript preparation. All authors have approved the final article prior to submission.

## Conflict of Interest Statement

The authors declare that the research was conducted in the absence of any commercial or financial relationships that could be construed as a potential conflict of interest.

## Abbreviations

CM, cutaneous melanoma; ICSEA, index of community socio-educational advantage; IQR, inter-quartile range; MN, melanocytic nevi; RCT, randomized controlled trial; SSS, SunSmart school; UVI, ultraviolet index; UVR, ultraviolet radiation.
